# What Factors Predict Falls in Older Adults Living in Nursing Homes: A Pilot Study

**DOI:** 10.3390/jfmk4010003

**Published:** 2018-12-25

**Authors:** Aditi Datta, Rahul Datta, Jeananne Elkins

**Affiliations:** 1 College of Professional Studies, Northeastern University, Boston, MA 02115, USA; 2 Mendel University In Brno. Zemědělská 1665/1, 613 00 Brno, Czech Republic

**Keywords:** fall, nursing home, gait speed, environmental cause

## Abstract

Background: In community-dwelling older adults, slow gait speed is linked to falls; however, little is known about the use of gait speed to predict falls in nursing home residents. The prevalence of risk factors for falls in nursing home residents is multifactorial. Objective: The purpose of this study was to examine the relationship between falls and multiple factors such as age, sex, gait speed, mobility device, fear of falling, cognitive function, medication, and environmental causes in a nursing home setting. Material and Methods: Participants were recruited from a nursing home. Independent variables such as age, sex, gait speed for 40 feet, use of a mobility device, fear of falls, cognitive function, medication, and environmental causes of falls were measured and recorded. The dependent variable was falls. Participants were followed-up for a period of six months for falls. Falls were documented from the computerized medical records at the facility. Results: Five of the 16 participants had falls in the follow-up period. Exact logistic regression, bivariate analysis, showed no significant relationship between falls and the independent variables of age, sex, gait speed, mobility device, fear of falls, cognitive function, and medication. More than 30% of recorded falls had an environmental cause, which was significant at *p* = 0.0005. Conclusion: Environmental causes had a significant relationship with falls in nursing home participants. Environment hazard monitoring is therefore important to ensure the safety of nursing home residents.

## 1. Introduction

Residents of nursing homes suffer falls at nearly twice the rate of persons living in the community. With more than a million nursing home residents in the U.S. [1], falls are a major health concern. Every year, 60% of nursing home residents fall [2]. Falls can result in serious fatal and nonfatal injuries, such as fractures, lacerations, or head injuries. Fall-related injuries, such as hip fractures, increase the mortality rate within the first six months [3]. Falls increase the financial burden on the U.S healthcare system for hospitalization and continued care as about 30% of persons who fall require medical attention [4]. In 2015, direct medical cost due to falls was estimated at $31 billion [5].

The prevalence of risk factors for falls in nursing home residents is multifactorial [6]. Both intrinsic and extrinsic risk factors contribute to falls. Increased age [7], fear of falling [8,9,10], cognition [11], mobility device usage [12], health status [13,14], the living environment [15,16], and medication [17] have all been linked to falls. Some of the risk factors are modifiable, including the living environment and medications, but others, such as increasing age, are not modifiable.

The unmodifiable risk factors of sex and age are primary predictors of nursing home falls. Along with increasing age, female sex is predictive of falls [8]. The risk of falling is higher in women compared to men [18]; moreover, women sustain 40% to 60% more injuries than men [18]. This may be because residents who are women make up more than two-thirds of the U.S. nursing home population [19].

Fear of falling, a potentially modifiable belief, is a substantial risk for falls. While 50% of older adults have never fallen, they fear falling [20,21]; furthermore, among older adults who have fallen, 40% to 73% fear of falling [22]. Compared to community dwellers, older adults residing in a nursing home are more fearful of falling, with 46% answering “yes” when asked: “Are you afraid of falling?” [23]. The use of mobility devices increases the risk of falls [24], but it often offers individuals independence that could not be attained without it. Thus, family members and healthcare professionals often recommend the use of a mobility device. The rate of injury from the use of a mobility device is highest among those aged 85 years and older. Each year, more than 47,000 older adults are treated in the emergency departments in the U.S. due to falls related to mobility devices [12]. Most of the injuries from using a mobility device involve walkers (87%) [12].

Cognitive impairment, the living environment, and medications also contribute to falls. These extrinsic factors can be modified but are unrecognized in fall risk analysis for nursing home residents. Cognitive impairment, linked to increased risk of fall in older adults, may be mild, moderate, or severe and stems from many causes [25]. More than 50% of patients in nursing homes have diagnosed cognitive impairment [26]; however, many people have undiagnosed dementia. Irrespective of formal diagnosis, persons with dementia are 4–5 times more likely to experience falls [27]. Major classes of medications, a reversible risk factor for falls in older adults, have been linked to falls in numerous studies. These medication classes include antipsychotics, antidepressants, and sedatives–hypnotics, particularly benzodiazepines [28]. Common side effects are sedation, impaired balance, and decreased coordination. While there has been a recent push to reduce the use of antipsychotics in nursing homes, nearly one-third of nursing home residents with dementia receive antipsychotic medications [29].

Environmental hazards can contribute to falls. These hazards include wet floors, poor lighting, unstable furniture, clutter, and absence of grab bars among other hazards. Environmental hazards account for 16% to 27% of falls in nursing homes [6]. Three out of four nursing home residents fall every year due to environmental causes [30]. Cognitively impaired older adults are at greater risk of falls due to decreased safety awareness to avoid hazards in their environment [27].

Gait speed has been associated with health status and functional decline [14], including risk of hospitalization and death [31]. In community-dwelling adults who are older, changes in gait speed has been associated with risk of falls [32] and adverse events [33]. As gait speed is simple, quick, inexpensive, and highly reliable [34], it can be routinely incorporated into clinical practice to assess the risk of falls in older adults. Slower gait speed of less than 0.6 m/s is predictive of unfavorable outcomes such as falls and hospitalization among community-dwelling older adult [35,36,37]. However, the efficacy of using gait speed to predict falls in nursing home residents is unknown. To date, little data exists to determine if slower gait speed is associated with falls in nursing homes. The aim of this pilot study is to examine the relationship between falls and multiple factors such as age, sex, gait speed, mobility device, fear of falling, cognitive function, medication, and environmental causes in older adults living in nursing homes.

## 2. Material and Methods

This study was approved by the Northeastern University Institutional Review Board (IRB), code #CPS15-08-23, on 22 September 2015. All participants were consented prior to participation. All participants resided in the same Medicare-certified skilled nursing facility in South Bend, IN, USA.

### 2.1. Subjects

Sixteen participants were recruited for the study. These participants met the following pre-established and IRB-approved study criteria: aged 65 years and above; ambulatory with or without assisted device; no history of CVA (cerebrovascular disease); no profound neuromuscular disease, musculoskeletal disease, Parkinson’s disease, or connective tissue disease; no severe visual impairment; and no limb amputation. Dementia was considered a diagnosis only if it was diagnosed by a physician and was part of the patient’s medical record. Out of the 16 participants recruited, 12 were females and 4 were males. Ten participants used a walker as an assisted device for walking. Self-rated health and fear of falling was measured before measuring gait speed. For self-rated heath, participants were instructed to circle or point on the test paper how they feel about their health today (0 = poor, 1 = fair, 2 = good, 3 = very good, 4 = excellent). For measuring fear of falling, all participants were simply asked to say “yes” or “no” to the question “Are you fearful of falling” during walking. All participants’ active medications were reviewed before recording gait speed (Table 1). 

Fall history of all participants from the day of measurement of gait speed up to a period of six months was obtained from the nursing facility’s computerized medical documentation records. (Table 2).

Of the participants, 75% were females (Table 3). As shown in Table 3, the mean age was 76 years with a range of 65–91 years. More than half (62.5%) of the participants used walker as a mobility device for ambulation. Among the participants, 68.75% were fearful of falling. Self-rated health was reported to be “very good” by 50%, “good” by 25%, “fair” by 18.75%, and excellent by 6.25% of participants. Half of the participants (50%) were cognitively impaired with a diagnosis of dementia. During the time of follow-up, five participants had falls. More than one-third (31.5%) of these falls were due to environmental causes.

### 2.2. Gait Speed Measurement

An earlier survey of the literature showed that gait speed tests vary in distance ranging from 4 to 500 m [38]. For this study, we selected a short distance of 40 feet, i.e., 12.19 m, on a level surface corridor with no turns. Participants were instructed to walk at their normal pace as they would walk every day in the corridors. Gait speed was measured by having participants walk 12.19 m at their normal pace with or without using an assisted device on a straight pathway marked with cones. Only one measurement was recorded. Time taken to walk 12.19 m was recorded using a stop watch. Gait speed was calculated as follows (1):

(1) $$Gait\;speed\;(m/s)=\frac{Distance}{Time}\frac{\left(m\right)}{\left(s\right)}$$

The slowest gait speed was 0.40 m per second, while the fastest gait speed was 1.12 m per second (Table 4).

## 3. Statistical Analysis

As this is a pilot study and our sample size was predicted to be small, we used exact logistic regression in STATA 13 (StataCorp, College Station, TX, USA) for analysis. We created bivariate exact logistic regression models to examine the relationship between the dependent variable (falls) and each independent variable (sex, age, mobility aid, fear of falling, self-rated health, medications, and environmental causes), as shown in Table 5. Statistical significance was set at an alpha level of 0.05.

## 4. Results and Discussion

In this study, independent variables of age, sex, mobility device, fear of falling, cognitive function, and medication did not show statistical significance with the dependent variable, i.e., fall. However, environmental causes reached significance of *p* = 0.005. Gait speed did not reach significance (*p* = 0.278) but did increase the odds of falls by 5.82 (OR 5.82, 95% CI: 0.40–123.27). The odds of falls for each unit increase in cognitive impairment increased by 6. Women were more likely to fall than men. The odds of fall for women increased by 2. The environmental causes of falls were significant in the bivariate model with falls as the dependent variable. In this model, environment causes increased odds of falls by 64 (OR 64.003, 95% CI: 5.566613 ± Inf) and was significant at 0.0005 (Table 5). 

**Figure 1:**
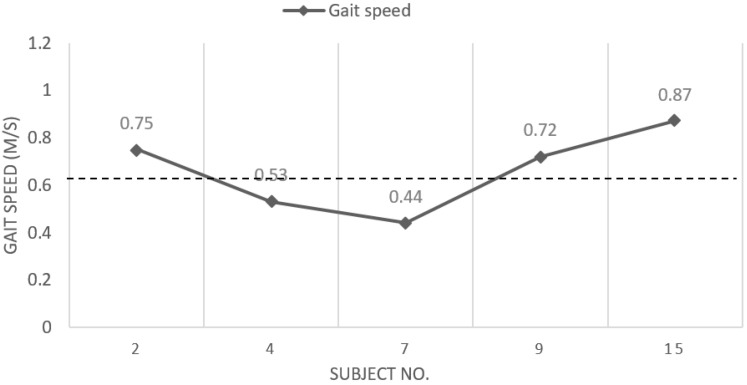
Gait speed of subjects with falls. The dashed line represents gait speed of 0.6 m/s, which is considered as the cutoff for predicting falls [35,36,37].

Out of the 16 participants who completed the study, five participants (subject no. 2, 4, 7, 9, and 15) had falls (Table 2; Figure 1). Among the five participants who fell, gait speed of subject no. 2 was highest (0.75 m/s), while gait speed of subject no. 7 was lowest (0.44 m/s) (Table 2). 

Fifteen participants had gait speed below the community-dwelling ambulators (Table 2). Five participants (subject no. 2, 4, 7, 9, 15) had falls (Table 2; Figure 1). Three participants with falls (subject no. 2, 9, 15) had gait speed above 0.6 m/s (Figure 1), which is considered as the cutoff value for predicting falls [35,36,37]. The results indicated that, in this study, slower gait speed was not significantly (*p* = 0.28) related to falls. Slow gait speed (<0.6 m/s) has been shown to be a predictor of falls in community-dwelling older adults [35,36,37]. However, gait speed was not a predictor of falls in our participants in the nursing home. During the detailed review of fall records, it was observed that the participants who fell had at least one fall due to an environmental hazard such as a wet floor, narrow doorways, clutter in the room, and tripping on door rails. Thus, we suggest that reducing environmental hazards should be considered important to prevent falls in nursing home residents. 

Our study had several limitations. The sample size was too small to detect meaningful changes in gait speed. This may have affected the association between slow gait speed and falls. The follow-up period of six months for subsequent falls was also short. Previous studies on gait speed have used 18 months or longer follow-up periods. Cognitive impairment was determined through the diagnosis of dementia by MD (Medical Director). The evaluation of cognitive impairment was not performed using neurological tests, such as Mini-Mental State Examination, and so the degree and stage of cognitive impairment were unknown. Nevertheless, this pilot study could be useful in conducting a larger study in the future among nursing home residents, and we strongly believe that our results will have significant impact on the interpretation of other studies done on larger sample sizes.

## 5. Conclusions

Although this was a pilot study, we could say from our results that the relationship between gait speed and falls in community-dwelling older people cannot be used in nursing home settings. We therefore suggest that other factors such as environmental hazards should also be considered in nursing home participants to predict falls. This study demonstrates the importance of reducing environmental hazards for fall prevention in nursing home residents

## Figures and Tables

**Table 1:** List of prescribed medications for all participants.

Subject No.	Falls	Antidepressant	Antianxiety	Antihypertensive	Diuretic	Β-Blockers	Statins	Antipsychotic
1	0				×	×		
2	3						×	
3	0		×	×				×
4	1						×	
5	0	×						
6	0							
7	6						×	×
8	0						×	×
9	1			×		×		
10	0	×			×	×		×
11	0							
12	0					×		×
13	0	×						×
14	0			×	×			
15	3	×	×					
16	0							×

**Table 2:** Demographic data, gait speed, and fall records.

No.	Sex	Date	Age	Time	Gait Speed (m/s)	Mobility Aid	Fear of Falling	Self-Rated Health	Falls
1	M	7/1/2015	65	17.47	0.70	walker	no	3	0
2	F	6/25/2015	70	16.35	0.75	walker	yes	2	3
3	F	6/25/2015	70	10.84	1.12	none	no	1	0
4	F	6/25/2015	85	23.06	0.53	walker	no	1	1
5	M	6/30/2015	90	20.15	0.61	walker	no	2	0
6	F	7/30/2015	90	23.42	0.52	walker	no	2	0
7	M	8/18/2015	90	27.66	0.44	walker	no	3	6
8	F	6/25/2015	78	15.05	0.81	none	no	3	0
9	F	9/24/2015	87	16.88	0.72	none	no	1	1
10	F	10/1/2015	83	20.3	0.60	walker	yes	3	0
11	F	10/15/2015	77	22.46	0.54	none	no	2	0
12	F	9/21/2015	73	20.01	0.61	none	no	3	0
13	F	11/9/2015	66	22.18	0.55	walker	yes	3	0
14	M	7/11/2015	85	19.46	0.63	walker	yes	3	0
15	F	8/15/15	74	13.97	0.87	none	no	3	3
16	F	11/9/2015	91	30.53	0.40	walker	yes	4	0

**Table 3:** Descriptive statistics. *n* = 16.

Variable	% (*n*)	Range	Mean
Sex			
Male	25% (4)		
Female	75% (12)		
Age		65–91	79.625
Mobility aid			
Yes	62.5% (10)		
No	37.5% (6)		
Fear of falling			
Yes	31.25% (5)		
No	68.75% (11)		
Self-rated health			
Fair	18.75% (3)		
Good	25.00% (4)		
Very Good	50.00% (8)		
Excellent	6.25% (1)		
Cognitive			
Diagnosed	50% (8)		
No	50% (8)		
Falls—reported and recorded	Total 5		
None	68.75% (11)		
One	12.50% (2)		
Three	12.50% (2)		
Six	6.25% (1)		
Environmental cause of fall			
Yes	100% (5/5)		
No	0% (0/5)		
Medications			
Antipsychotics	43.75% (7)		
Antidepressants	25% (4)		

**Table 4:** Gait peed. *n* = 16.

Variable	
Time for walking 12.19 m	s
Maximum	30.53
Minimum	10.94
Gait speed	m/s
Fastest	1.12
Slowest	0.40

**Table 5:** Results of bivariate analysis: falls as dependent variable. *n* = 16; * statistical significance set at α = 0.05.

Variable	Odds Ratio	*p*-Value	95% Confidence Interval
Sex	1.464	1.000	0.080707–98.16351
Age	0.866	1.000	0.0640751–14.65901
Gait speed	0.977	0.846	0.7774365–1.211467
Mobility aid	0.866	1.000	0.0640751–14.65901
Fear of falling	0.459	0.967	0.0071286–7.359933
Self-rated health	0.438	0.315	0.080355–1.763968
Cognitive status	6.157	0.282	0.4139234–392.7872
Environmental cause of fall	64.003	0.0005 *	5.566613 ± Inf
Medications	1	-	
